# Meta-Analysis on Randomized Controlled Trials of Vaccines with QS-21 or ISCOMATRIX Adjuvant: Safety and Tolerability

**DOI:** 10.1371/journal.pone.0154757

**Published:** 2016-05-05

**Authors:** Emilia Bigaeva, Eva van Doorn, Heng Liu, Eelko Hak

**Affiliations:** 1 Department of Pharmaceutical Technology and Biopharmacy, University of Groningen, Groningen, The Netherlands; 2 Department of Pharmacy, Unit of PharmacoEpidemiology & PharmacoEconomics (PE2), University of Groningen, Groningen, The Netherlands; Centers for Disease Control and Prevention, UNITED STATES

## Abstract

**Background and Objectives:**

QS-21 shows *in vitro* hemolytic effect and causes side effects *in vivo*. New saponin adjuvant formulations with better toxicity profiles are needed. This study aims to evaluate the safety and tolerability of QS-21 and the improved saponin adjuvants (ISCOM, ISCOMATRIX and Matrix-M^™^) from vaccine trials.

**Methods:**

A systematic literature search was conducted from MEDLINE, EMBASE, Cochrane library and Clinicaltrials.gov. We selected for the meta-analysis randomized controlled trials (RCTs) of vaccines adjuvanted with QS-21, ISCOM, ISCOMATRIX or Matrix-M^™^, which included a placebo control group and reported safety outcomes. Pooled risk ratios (RRs) and their 95% confidence intervals (CIs) were calculated using a random-effects model. Jadad scale was used to assess the study quality.

**Results:**

Nine RCTs were eligible for the meta-analysis: six trials on QS-21-adjuvanted vaccines and three trials on ISCOMATRIX-adjuvanted, with 907 patients in total. There were no studies on ISCOM or Matrix-M^™^ adjuvanted vaccines matching the inclusion criteria. Meta-analysis identified an increased risk for diarrhea in patients receiving QS21-adjuvanted vaccines (RR 2.55, 95% CI 1.04–6.24). No increase in the incidence of the reported systemic AEs was observed for ISCOMATRIX-adjuvanted vaccines. QS-21- and ISCOMATRIX-adjuvanted vaccines caused a significantly higher incidence of injection site pain (RR 4.11, 95% CI 1.10–15.35 and RR 2.55, 95% CI 1.41–4.59, respectively). ISCOMATRIX-adjuvanted vaccines also increased the incidence of injection site swelling (RR 3.43, 95% CI 1.08–10.97).

**Conclusions:**

Our findings suggest that vaccines adjuvanted with either QS-21 or ISCOMATRIX posed no specific safety concern. Furthermore, our results indicate that the use of ISCOMATRIX enables a better systemic tolerability profile when compared to the use of QS-21. However, no better local tolerance was observed for ISCOMATRIX-adjuvanted vaccines in immunized non-healthy subjects. This meta-analysis is limited by the relatively small number of individuals recruited in the included trials, especially in the control groups.

## Introduction

Adjuvants are substances that do not confer immunity on their own [1]. However, when added to immunogens, they facilitate, improve and maintain the immune responses against the immunogens [2,3]. Therefore, adjuvants provide a rational strategy to improve the efficacy of vaccines, especially in the case of weak immunogens and/or vaccines intended for individuals with weakened immune systems (e.g. newborns, the elderly or immune-compromised persons). Furthermore, adjuvants allow for dose sparing of vaccine antigen and helps in reducing the cost of vaccination programs [4,5].

A group of immunoenhancers of great interest is saponins, whose strong adjuvant activity was first described in 1930s [6]. Saponins are natural triterpenoid or steroid glycosides, which can be extracted from the bark of a South American tree *Quillaja saponaria* (Soapbark) [7,8]. QS-21 is one of the most potent and the most extensively studied saponin adjuvants in numerous studies including prophylactic and therapeutic vaccines for both animals and humans [9,10]. QS-21 has been shown to be an effective immunological adjuvant for human vaccines with a wide variety of antigens and to have a relatively low toxicity in preclinical studies in mice [11,12]. It stimulates both antibody and cellular immune responses composed of both Th1 and Th2 immunity. The cellular immune stimulating capacity of QS-21 is especially important for developing vaccines against cancers and intracellular pathogens [13]. A number of vaccine trials have been performed using QS-21 as adjuvant, initially for cancer vaccines (i.e. melanoma, breast and prostate cancer) and, subsequently, for vaccines against Alzheimer’s disease and infectious diseases, including human immunodeficiency virus (HIV)-1, influenza, herpes simplex virus (HSV), malaria and hepatitis B diseases [9,12].

However, the natural saponin QS-21 has inherent disadvantages such as chemical instability, limited supply, difficult and low-yielding purification, and dose-limiting toxicity, which prevent it from wider use [14]. Importantly, QS-21 could cause hemolysis *in vitro* and its use *in vivo* has been observed with side effects [7]. Saponins have been shown to interact with cholesterol, and might form pores in the lipid bilayer of cell membranes. When such interaction happens to erythrocytes, hemolysis could occur [15].

In order to reduce saponin-related toxicity, the formulation of immunostimulating complex (ISCOM) was developed by Morein *et al*. in 1984 [16]. ISCOMs are open cage-like 40-nm particulate structures, which are formed spontaneously if cholesterol, phospholipids, saponin and viral envelope proteins are mixed together [17,18]. The formulation retains the adjuvant activity of the saponin with an increased stability, when compared to QS-21. Furthermore, the concern of hemolysis is solved by eliminating the possibility of the saponin to interact with cell membranes [19,20]. Nevertheless, the types of antigens that can be incorporated into ISCOM are restricted, and the incorporation process is difficult to control [4].

Due to these technical problems and the fact that antigen incorporation is not necessary to achieve a potent immune stimulation, matrix formulations such as ISCOMATRIX and Matrix-M^™^ were developed. These matrix formulations contain the same components and have the same structure as the ISCOM but without the incorporated antigen [19,21]. ISCOMATRIX usually contains Quil A or more purified forms of saponins, including QS-21, ISCOPREP^™^ 703 and, more recently, ISCOPREP [22]. Matrix-M^™^ is a combination of two individually formed matrix particles from different purified fractions of Quillaia saponins, namely Matrix-A^™^ and Matrix-C^™^ [23]. The former fraction has a higher adjuvant activity while the latter fraction has a lower adjuvant activity but a high tolerance [19].

Both ISCOMATRIX and Matrix-M^™^ adjuvanted vaccines have been tested in animal models and more recently in human clinical trials [19,22,23]. Vaccines adjuvanted with either ISCOMATRIX or Matrix-M^™^ have been shown to induce strong antibody and T-cell responses and to be well tolerated in both pre-clinical and clinical studies [24,25]. ISCOMATRIX is currently under evaluation in candidate vaccines against hepatitis C virus (HCV) [26], influenza [27] and cancer [28–30]. Matrix-M^™^ is currently being investigated in vaccines for influenza, HSV type 2 and malaria [23,31]. Current applications of ISCOMs include the development of influenza vaccines for humans [32].

The benefits from adjuvant incorporation into any vaccine formulation have to be balanced with the risk of adverse events (AEs) [2]. The purpose of this meta-analysis is to evaluate the safety and tolerability of QS-21 and the improved saponin-based adjuvants such as ISCOMATRIX. Here we focus on single adjuvant formulations (QS-21, ISCOM, ISCOMATRIX and Matrix-M^™^) rather than combinations of adjuvants. Therefore, studies that used adjuvant systems, such as AS01, AS02 and AS04 developed by GlaxoSmithKline, were not included in the analysis.

## Methods

We conducted a meta-analysis according to the Preferred Reporting Items for Systematic Reviews and Meta-Analyses (PRISMA) statement [33]. The protocol for the study was not published online.

### Literature search strategy

A systematic search of literature was performed using the electronic databases of MEDLINE (Ovid), EMBASE and Cochrane Central register of Controlled Trials (CENTRAL). The clinical trial register (clinicaltrials.gov) was searched for unpublished trials. To define the studies of interest in these databases, the following keywords were used: “QS-21”, “ISCOMs”, “ISCOMATRIX”, “Matrix-M”, “randomized controlled trial” and “clinical trial”. Details of the search strategy are provided in the supporting information (S1 Appendix). References were imported to RefWorks where duplicate entries were removed. Furthermore, the literature search was complemented by manual search of the reference lists of all identified studies and reviews for additional studies.

### Study selection

References were evaluated using the pre-defined inclusion criteria: (1) randomized controlled trials (RCTs) on vaccines with saponin adjuvants (QS-21, ISCOM, ISCOMATRIX or Matrix-M^™^); (2) which included a control group (i.e., individuals immunized with saline buffer, adjuvant alone, antigen alone or adjuvanted with a licensed adjuvant); and (3) reporting information regarding safety and/or tolerability. The inclusion of only RCT studies was considered necessary to avoid the possible selection and reporting biases that may arise from observational studies.

Two independent reviewers (EB, ED) performed primary evaluation of the retrieved articles for relevance based on the title and abstract. Disagreements were discussed with a third investigator (HL) until consensus was achieved. Potentially eligible publications were reviewed as full text. The acronym PICOS (patients, interventions, comparator (control) group, outcomes and study design) was used to assess if the references fully complied with the inclusion criteria. In order to lower the between-study heterogeneity and due to the fact that there were not enough eligible RCTs in healthy volunteers to be included for the meta-analysis, we limited our study selection to RCTs that recruited adult (18 years and older) non-healthy subjects. References for which full-text could not be acquired electronically or were reported not in English language were excluded.

### Data extraction

Two independent reviewers (EB and ED) identified potentially relevant articles and collected the following data: the first author’s last name, the year of publication, clinicaltrials.gov identifier (if applicable), study design, total number of participants, age range, gender, disease background, study arms with number of vaccinated participants in each arm, doses of adjuvants used for the preparation of vaccines, immunization route and number of injections.

The following safety outcomes were identified from the included studies and considered for the meta-analysis: serious, systemic and local AEs. The commonly reported systemic AEs across the selected studies included headache, fatigue, insomnia, pyrexia, myalgia, nausea, diarrhea, dizziness, anxiety and back pain. The local AEs included injection site pain, redness, erythema and swelling.

### Evaluation of study quality

Following Cochrane guidelines for systematic reviews of interventions [34], two independent reviewers (EB, ED) assessed the quality of individual studies included in the meta-analysis. The Jadad scale for reporting RCTs, which summarizes the methodological quality of a study in an overall score, was employed. In brief, the Jadad scale evaluates three items: randomization (up to two points are given), double blinding (up to two points are given) and report of withdrawals and dropouts (up to one point is given). An overall score between zero and five is assigned. A score of three and above is commonly regarded as the reference point for adequate trial quality [35]. Studies were not to be excluded on the basis of this assessment but their quality scores were taken into account when describing results.

### Data analysis

To evaluate the safety and tolerability of saponin adjuvanted vaccines, the dichotomous data on the number of subjects experiencing a systemic or local AE in the saponin-adjuvanted study vaccine group and placebo group were extracted from each study with subsequent determination of the risk ratios (RR) and their 95% confidence intervals (CI). Of note, within each study we pooled all subjects that received adjuvanted vaccine, regardless the concentration of the adjuvant and antigen in vaccine formulation. We combined data statistically using random effects (Mantel-Haenszel method) model due the differences in among the studies in e.g. vaccine formulation, adjuvant dose and the disease background of subjects. Chi^2^ and *I*^*2*^ statistics were used to assess the heterogeneity among the included studies. Values of *I*^*2*^ can be interpreted as low (25–50%), moderate (50–75%), and high (75% and greater) levels of heterogeneity [36]. Meta-analyses were performed using Review Manager (RevMan 5.3, Cochrane Collaboration). Results were considered to be statistically significant with a *p* value of <0.05. In addition to the meta-analysis, descriptive reports on serious adverse events (SAEs) and treatment discontinuations were given.

### Dealing with missing data

Our analysis relies solely on the existing data.

### Assessment of reporting biases

Due to the limited number of studies available for meta-analysis, assessment of publication bias was not applicable. The review is subject to publication bias.

## Results

### Search results

**Fig 1:**
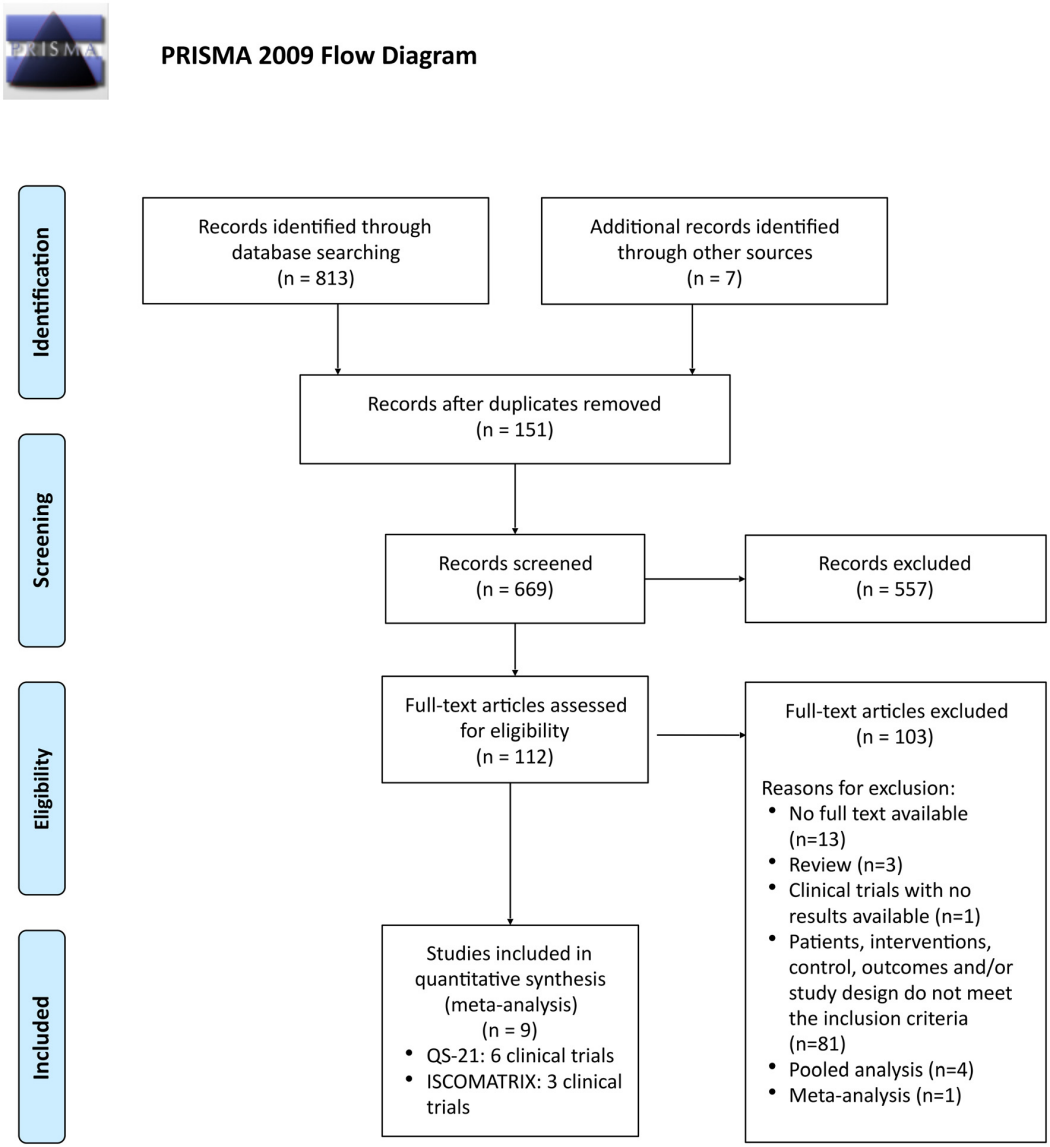
Flowchart of the evaluation and inclusion process for the meta-analysis. *From*: Moher D, Liberati A, Tetzlaff J, Altman DG, The PRISMA Group (2009). *P*referred *R*eporting *I*terns for *S*ystematic Reviews and *M*eta-*A*nalyses: The PRISMA Statement. PLoS Med 6(6): e1000097. doi:10.1371/journal.pmed1000097 For more information, visit www.prisma-statement.org.

A total of 813 references were identified from electronic databases during the search performed during 03–04.03.2016 (Fig 1). Additional 7 references were identified by manual search. After removing duplicate entries (151), 669 references were evaluated for inclusion based on the title and/or abstract. As a result, 112 potentially relevant articles were included in the next stage for the full-text evaluation. From the 112 articles, full text was unavailable for 13 studies, 3 references were reviews, and 1 reference was a completed clinical trial with no study results reported. Characteristics of the study population, interventions, control groups, the evaluated outcomes and/or design of the study (PICOS) did not meet the inclusion criteria in 81 publications. Most of these studies did not include a control group, i.e. all enrolled subjects received the saponin-adjuvanted vaccines. One meta-analysis [37] and four pooled analyses [22,38–40] were excluded since the original safety data of the reported studies were not retrievable. Ultimately, a total of nine RCTs fulfilled all inclusion criteria and were selected for the meta-analysis [41–49].

### Study characteristics

The main characteristics of the selected RCTs are summarized in Table 1. Among nine studies, six used QS-21 and three used ISCOMATRIX as vaccine adjuvant. No RCTs on ISCOM or Matrix-M^™^ adjuvanted vaccines matched the inclusion criteria. Briefly, the trials on ISCOM (one trial) and Matrix-M^™^ (one trial [50], two reports [51,52]) adjuvanted vaccines were performed in healthy volunteers and/or did not report safety data.

**Table 1:** Characteristics of the included studies.

Author, year	Enrollment	Age range (yrs)	M:F	Health status	Study arms	Adjuvant dose	Route/ N° of vaccin	Ref
**QS-21**								
Gilman et al, 2005	372	50–85	167:205	AD	*Arm1*: AN1792 225 μg + QS-21 (n = 300)	50 μg	IM/6	[41]
					*Arm2*: Placebo (n = 72)			
Wald et al, 2011	35	21–54	11:24	HSV-2	*Arm1*: HerpV 80 μg + QS-21 (n = 7)	50 μg	SC/3	[42]
					*Arm2*: HerpV 240 μg + QS-21 (n = 6)			
					*Arm3*: HerpV 80 μg (n = 6)			
					*Arm4*: HerpV 240 μg (n = 6)			
					*Arm5*: QS-21 (n = 5)			
					*Arm6*: Placebo (n = 5)			
Pfizer, 2014^#^	245	60–78	106:139	AD	*Arm1*: ACC-001 3 μg + QS-21 (n = 36)	50 μg	IM/5	[43,44]
(NCT00479557)	(86)				*Arm2*: ACC-001 10 μg + QS-21 (n = 61)			
(NCT00498602)	(159)				*Arm3*: ACC-001 30 μg + QS-21 (n = 40)			
					*Arm4*: ACC-001 10 μg (n = 35)			
					*Arm5*: ACC-001 30 μg (n = 12)			
					*Arm6*: QS-21 50 μg (n = 44)			
					*Arm7*: Placebo (n = 17)			
Pfizer, 2014 (a)	40	62–77	17:23	AD	*Arm1*: ACC-001 3 μg + QS-21 (n = 6)	50 μg	IM/5	[45]
(NCT00752232)					*Arm2*: ACC-001 10 μg (n = 6)			
					*Arm3*: ACC-001 10 μg + QS-21 (n = 6)			
					*Arm4*: ACC-001 30 μg (n = 6)			
					*Arm5*: ACC-001 30 μg + QS-21 (n = 6)			
					*Arm6*: QS-21 50 μg (n = 6)			
					*Arm7*: Placebo (n = 4)			
Pfizer, 2015	63	61–75	30:33	AD	*Arm1*: ACC-001 3 μg + QS-21 (n = 22)	50 μg	IM/6	[46]
(NCT01227564)					*Arm2*: ACC-001 10 μg + QS-21 (n = 20)			
					*Arm3*: Placebo (n = 21)			
**ISCOMATRIX**								
Anderson et al, 2009	35	35–58	35:0	HIV	*Arm1*: HPV16 E6E7 25 μg + IMX (n = 4)	120 μg	IM/3	[47]
					*Arm2*: HPV16 E6E7 70 μg + IMX (n = 8)			
					*Arm3*: HPV16 E6E7 240 μg + IMX (n = 8)			
					*Arm4*: HPV16 E6E7 240 μg + IMX (n = 8)			
					*Arm5*: Placebo (n = 7)			
Frazer et al, 2004	31	19–57	0:31	CIN	*Arm1*: HPV16 E6E7 20 μg + IMX (n = 9)	100 μg	IM/3 or 1	[48]
					*Arm2*: HPV16 E6E7 60 μg + IMX (n = 10)			
					*Arm3*: HPV16 E6E7 200 μg + IMX (n = 5)			
					*Arm4*: Placebo (n = 7)			
Sharp & Corp, 2012	86	65–84	41:45	AD	*Arm1*: V950 0.5 μg + IMX 16 μg (n = 8)	16, 47 or	IM/3	[49]
(NCT00464334)					*Arm2*: V950 5 μg + IMX 16 μg (n = 8)	94 μg*		
					*Arm3*: V950 50 μg + IMX 16 μg (n = 8)			
					*Arm4*: V950 0.5 μg + IMX 47 μg (n = 7)			
					*Arm5*: V950 5 μg + IMX 47 μg (n = 8)			
					*Arm6*: V950 0.5 μg + IMX 94 μg (n = 7)			
					*Arm7*: V950 0.5 μg (n = 8)			
					*Arm8*: V950 5 μg (n = 9)			
					*Arm9*: V950 50 μg (n = 5)			
					*Arm10*: IMX 16 μg (n = 13)			
					*Arm11*: Placebo (n = 5)			

*We included in our meta-analysis all intervention study arms (arm1-6), for which individuals received vaccines containing ISCOMATRIX (16, 47 or 94 μg) for a larger sample size.

^#^We combined two clinical trials sponsored by Pfizer (NCT00479557 and NCT00498602) since only pooled data from these two studies were disclosed.

**Abbreviations:** M, male; F, female; HIV, Human Immunodeficiency Virus; CIN, Cervical Intraepithelial Neoplasia; AD, Alzheimer’s Disease; HSV-2, genital Herpes Simplex Virus type 2; IMX, ISCOMATRIX; IM, Intramuscular; SC, Subcutaneously.

The six trials on QS-21-adjuvanted vaccines enrolled a total of 755 individuals, of which 510 in the treatment group (i.e. subjects received antigen with adjuvant) and 245 in the control groups (i.e. subjects received placebo, antigen alone or adjuvant alone). A total of 152 non-healthy subjects were recruited for the three RCTs on ISCOMATRIX-adjuvanted vaccines and included 98 and 54 in the treatment and control groups, respectively. The selected trials for both adjuvants recruited adult non-healthy subjects. Studies that involved healthy volunteers could not be included for the meta-analysis due to the limited number of identified studies that fulfilled the pre-defined inclusion criteria. The age of the enrolled subjects in the nine studies varied from 19 to 85 years, among which 44.9% were males.

All studies reported the number of subjects experiencing a specific AE. Studies of Anderson *et al*. [47] and Frazer *et al*. [48] used a seven-day diary card to record specific local and systemic AEs. In the former study, unsolicited AEs could also be reported on a separate 30-day diary card. In the study of Frazer *et al*. [48], an additional home visit was conducted with each study subject at the end of the follow-up period. Studies described by Gilman *et al*. [41] and Wald *et al*. [42] did not mention the method of AE reporting; however, they used physical examinations and evaluations of clinical and laboratory parameters after each vaccination. In the Sharp&Corp study [49], the safety data was collected up to 4 years after first dose of vaccine by systematic assessment (not further specified). During Pfizer studies NCT00479557 [43] and NCT00498602 [44] AEs were reported throughout 110 weeks, including a 6-week screening period, 52 weeks of dosing and 54 weeks for follow-up after the last dose. The studies NCT00752232 [45] and NCT01227564 [46] recorded AEs from day 1 throughout the trial (24 months and 104 weeks, respectively). All Pfizer studies used non-systematic assessment of AEs. Furthermore, trials NCT00479557, NCT00498602, NCT01227564 and Sharp&Corp (but not NCT00752232) used 5% frequency threshold for reporting AEs (not including SAEs). A threshold of 5% indicates that only AEs with a frequency greater than 5% within at least one arm were reported.

### Study quality

The methodological quality of the included RCTs was satisfying (Table 2), except for the study from Wald *et al*. [42] According to the Jadad scale, eight out of the nine studies (88.8%) have a score of 3 or 4. The study published by Wald *et al*. [42] has a score of 2 due to the fact that this RCT was single-blinded, and there was insufficient information on the randomization method.

**Table 2:** Quality assessment of included RCTs using Jadad scale.

	Gilman et al, 2005	Wald et al, 2011	Pfizer, 2014	Pfizer, 2014(a)	Pfizer, 2015	Anderson et al, 2009	Frazer et al, 2004	Sharp&Corp, 2012
Described as randomized*	1	1	1	1	1	1	1	1
Described as double-blind*	1	0	1	1	1	1 ^#^	1^#^	1
Description of withdrawals*	0	1	1	1	1	1	1	1
Randomization method described and appropriate**	1	0	0	0	0	1	-1	0
Double-blinding method described and appropriate**	0	0	0	0	0	0	1	0
**Score**	**3**	**2**	**3**	**3**	**3**	**4**	**3**	**3**

* A study receives a score of 1 for “yes” and 0 for “no”

** A study receives a score of 0 if no description is given, 1 if the method is described and appropriate, and -1 if the method is described but inappropriate.

^#^ The word “double-blind” was not used by the authors. However, according to the description of the blinding of the investigator, investigational site staff, and participants, one point was given for “described as double-blind”.

### QS-21-adjuvanted vaccine versus placebo

#### Serious adverse events (SAEs)

Wald *et al*. [42] reported no SAEs related to any treatment. On the other hand, treatment-related SAEs including encephalitis, encephalopathy, confusion, grand mal convulsion, retinal vein thrombosis, cerebral hemorrhage and hemiplegia were reported by Gilman *et al* [41]. The SAEs were observed in 7.3% (95% CI 4.9–10.8%) of QS-21-adjuvanted vaccine recipients and in 0% (95% CI 0–5%) of placebo recipients. This clinical trial was discontinued because of severe AEs, mostly associated with encephalitis. All subjects who reported meningoencephalitis received QS-21-adjuvanted vaccine. Deaths occurred during the follow-up period of the trial, however, with a similar incidence rate in the QS-21-adjuvanted vaccine group (1.7%, 95% CI 0.7–3.9%) and the placebo group (2.8%, 95% CI 0.8–9.6%). Of note, deaths in QS-21-adjuvanted vaccine group were caused by myocardial infarction, broken neck, progression of AD, or non-hemorrhagic cerebral infarct; whereas deaths in the placebo group were caused by neoplasm or cerebral hemorrhage.

All Pfizer studies [43–46] reported on SAEs. The SAEs incidence rate observed in the QS-21-adjuvanted vaccine groups was similar across the four trials (Pfizer 2014: 17.7%, 95% CI 12.2–24.9%; Pfizer 2014(a): 11.1%, 95% CI 3.1–32.1%; Pfizer 2015: 16.7%, 95% CI 8.3–30.6%). Furthermore, three out of four trials [43,44,46] showed that SAEs occurred with the similar or higher frequency in the placebo group (Pfizer 2014: 17.7%, 95% CI 6.2–41.0%; Pfizer 2015: 28.6%, 95% CI 13.8–49.9%). In contrast, Pfizer 2014(a) trial [45] reported no SAEs in the placebo group (0%, 95% CI 0–48.9%). The most common SAEs across the four Pfizer studies were: confusion, hallucinations, syncope, urinary tract infection, cardiac arrest, chest pain and hypotension. The pooled data from two Pfizer 2014 trials [43,44] indicated one case of death in the QS-21-adjuvanted vaccine group (the cause was not further specified). No deaths occurred during Pfizer 2014(a) and 2015 trials [45,46].

#### Systemic adverse events

**Fig 2:**
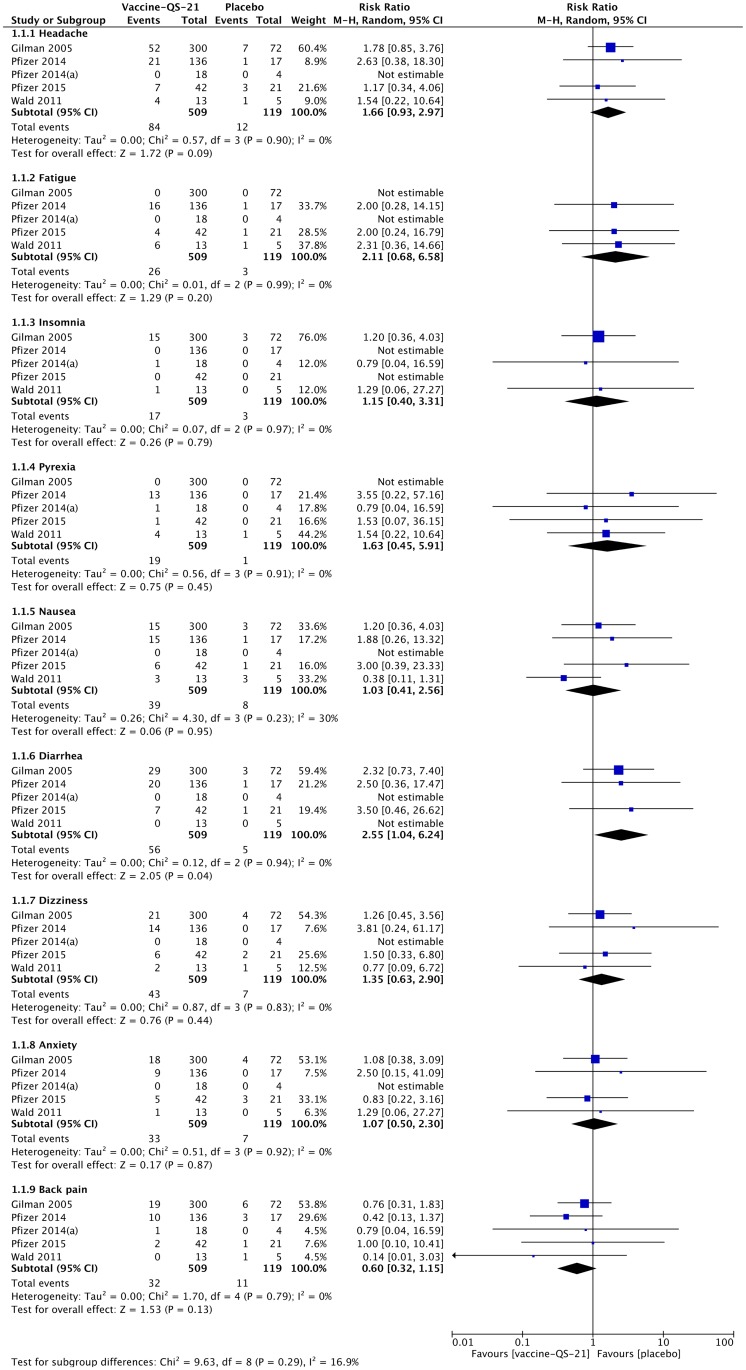
Meta-analysis of the reported systemic adverse events observed in non-healthy subjects receiving QS-21-adjuvanted vaccines or placebo.

The most frequent systemic AEs observed across the six studies include headache, fatigue, insomnia, pyrexia, nausea, diarrhea, dizziness, anxiety and back pain. The results of the meta-analysis demonstrated that out of the nine systemic AEs selected for the analysis, only cases of diarrhea were significantly more frequent in non-healthy subjects receiving QS-21-adjuvanted vaccines than in those receiving placebo (pooled RR 2.55, 95% CI 1.04–6.24, p = 0.04) (Fig 2). Furthermore, although the pooled RRs did not reach statistical significance, a trend towards a higher incidence of headache was observed in the QS-21-adjuvanted vaccine group (pooled RR 1.66, 95% CI 0.93–2.97, p = 0.09). Aside from the systemic AEs included in the meta-analysis, other commonly reported systemic AEs (≥ 5% of participants) were vomiting, myalgia, asthenia, upper respiratory tract infection, urinary tract infection, constipation, contusion and nasopharyngitis.

#### Local adverse events

Regarding the local AEs, we were not able to retrieve dichotomous data from RCT reported by Gilman *et al*. [41] However, the authors mentioned that injection site reactions were reported in both QS-21-adjuvanted vaccine group and placebo group, and the reported cases tended to be more severe and of longer duration among subjects treated with QS-21-adjuvanted vaccine. Furthermore, Pfizer 2015 trial [46] was not included in the meta-analysis on local AEs due to the fact that the only reported local AEs were injection site hematoma (7.1%, 95% CI 2.5–19.0% in QS-21-adjuvanted vaccine group; 0%, 95% CI 0–15.5% in placebo group) and injection site pruritus (4.8%, 95% CI 1.3–15.8% in QS-21-adjuvanted vaccine group; 0%, 95% CI 0–15.5% in placebo group).

**Fig 3:**
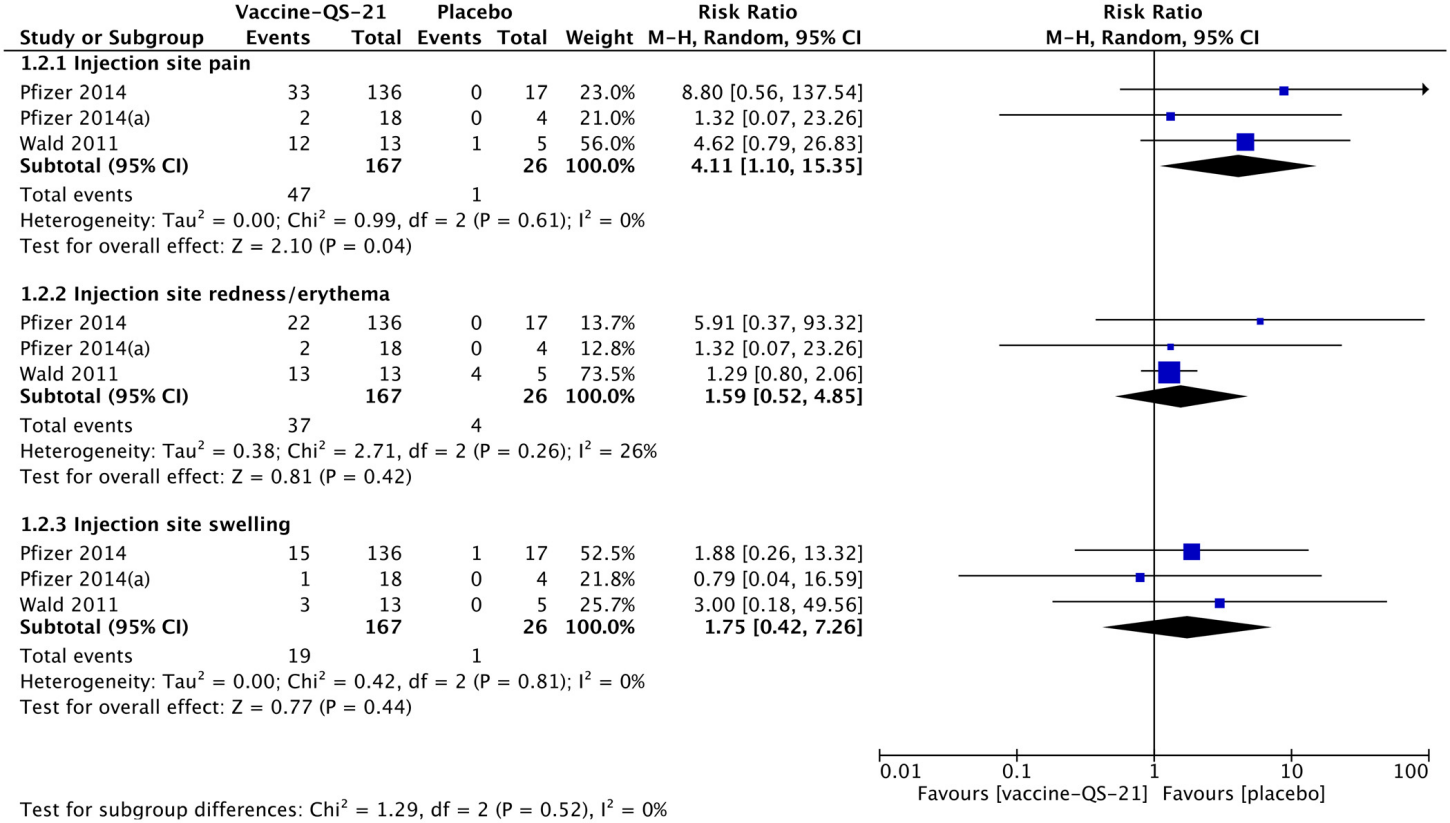
Meta-analysis of the reported local adverse events observed in non-healthy subjects receiving QS-21-adjuvanted vaccines or placebo.

The meta-analysis showed that QS-21-adjuvanted vaccines caused significantly more cases of injection site pain (pooled RR 4.11, 95% CI 1.10–15.35, p = 0.04) than placebo (Fig 3). No statistically significant increase in the risk for injection site redness/erythema and injection site swelling was observed with the immunization with QS-21-adjuvanted vaccines.

#### Discontinuations due to AEs

Together with SAEs, systemic and local AEs, we were interested in the cases of discontinuations due to the AEs. Wald *et al*. [42] reported that two subjects (5.7%, 95% CI 1.6–18.6%) dropped out form the study due to AEs that occurred in a temporal relation with the QS-21-adjuvanted vaccine. One subject developed severe arthralgia, and another developed mild neck pain and neck vein distention. The number of subjects who discontinued treatment due to AEs could not be retrieved from the study of Gilman *et al* [41]. However, the authors stated that the AEs leading to treatment discontinuations were more frequent among the participants receiving QS-21-adjuvanted vaccine than those receiving placebo. The two Pfizer 2014 trials [43,44] resulted in 4 dropouts in QS-21-adjuvanted vaccine group (2.9%, 95% CI 1.2–7.3%) due to the AEs related to the study vaccine with no cases of discontinuation in the placebo group (0%, 95% CI 0–18.4%). Furthermore, other 4 subjects discontinued the treatment in QS-21-adjuvanted vaccine group due to the AEs unrelated to the study vaccine. The AEs that caused discontinuation were not specified. No withdrawals due to AEs were reported from the Pfizer 2014(a) trial [45]. In general, these three trials showed higher incidence of discontinuations in the QS-21-adjuvanted vaccine group compared to the placebo. Pfizer 2015 trial [46] resulted in one case of discontinuation due to the AEs in QS-21-adjuvanted vaccine group and no cases in the placebo group. However, the total incidence of dropouts in Pfizer 2015 trial was twice higher in placebo group (28.6%, 95% CI 13.8–49.9%) than in QS-21-adjuvanted vaccine group (14.3%, 95% CI 6.7–27.9%).

### ISCOMATRIX-adjuvanted vaccine versus placebo

#### Serious adverse events

Studies of Anderson *et al*. [47] and Frazer *et al*. [48] reported no SAEs in ISCOMATRIX-adjuvanted vaccine recipients. The study from Sharp&Corp [49] detected SAEs in both treatment and control groups, and the observed SAEs included syncope, transient ischemic attack, squamous cell carcinoma of skin, and contusion. SAEs occurred in 19.6% (95% CI 10.7–33.2%) and 20% (95% CI 3.6–62.0%) of the recipients of the tested vaccine containing ISCOMATRIX and placebo, respectively. The same study reported a SAEs rate of 31.8% (95% CI 16.4–52.7%) in subjects who received only the antigen, and 0% (95% CI 0–22.8%) in those received only adjuvant (i.e. 16 μg ISCOMATRIX).

#### Systemic adverse events

**Fig 4:**
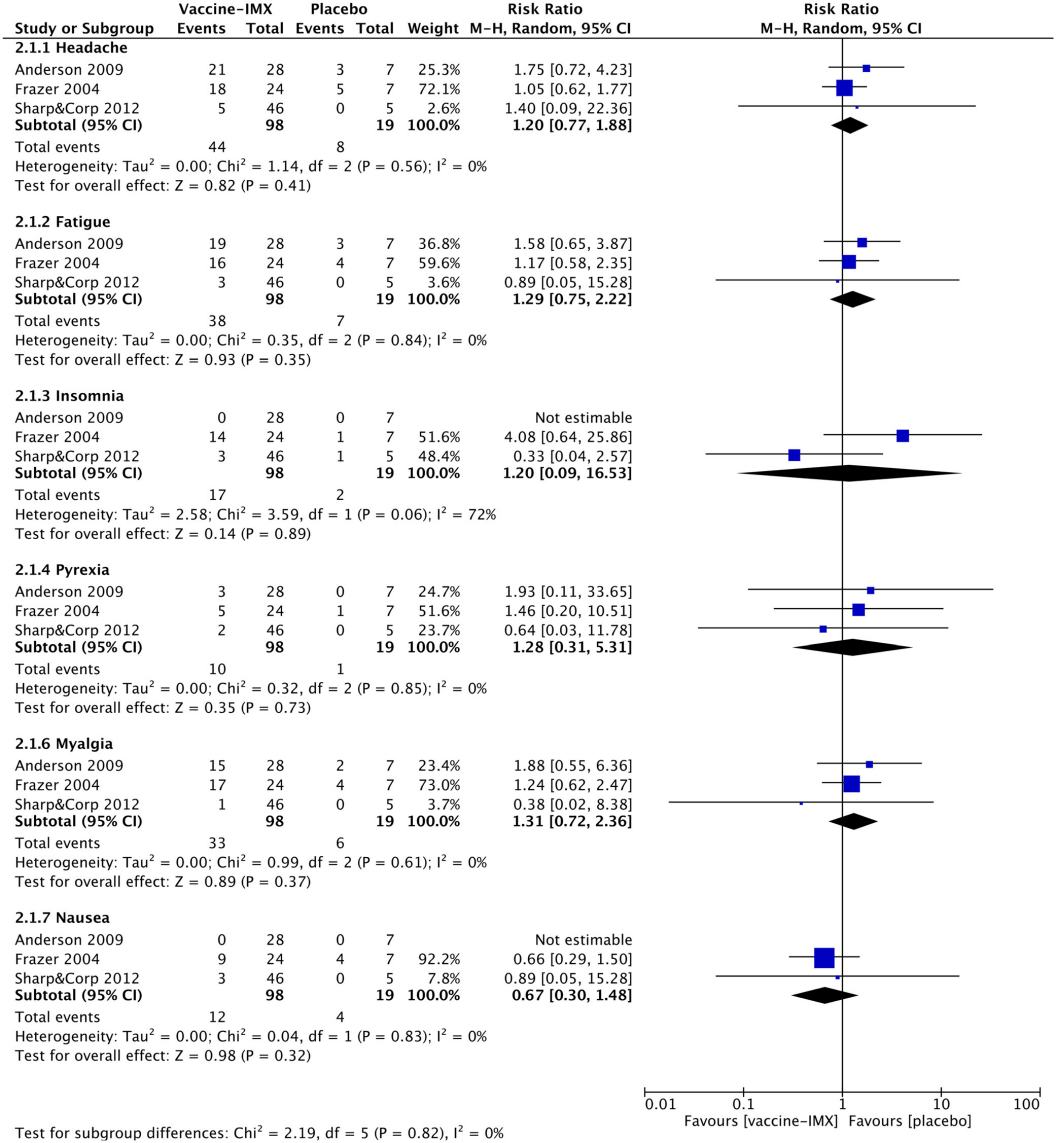
Meta-analysis of the reported systemic adverse events observed in non-healthy subjects receiving ISCOMATRIX-adjuvanted vaccines or placebo.

The most commonly reported systemic AEs across the selected studies include headache, fatigue, pyrexia, nausea, myalgia and insomnia. In general, the observed systemic AEs were mild to moderate in severity and lasted for 2–3 days. The proportion of subjects reporting systemic AEs was greater in the ISCOMATRIX-adjuvanted vaccine group than in the placebo group. The meta-analysis comparing the incidence of systemic AEs between the ISCOMATRIX adjuvanted vaccine group and placebo group showed no statistically significant differences in any selected for the analysis systemic AE (Fig 4). Furthermore, pooled RRs were generally lower for ISCOMATRIX-adjuvanted vaccines than for vaccines containing QS-21.

#### Local adverse events

**Fig 5:**
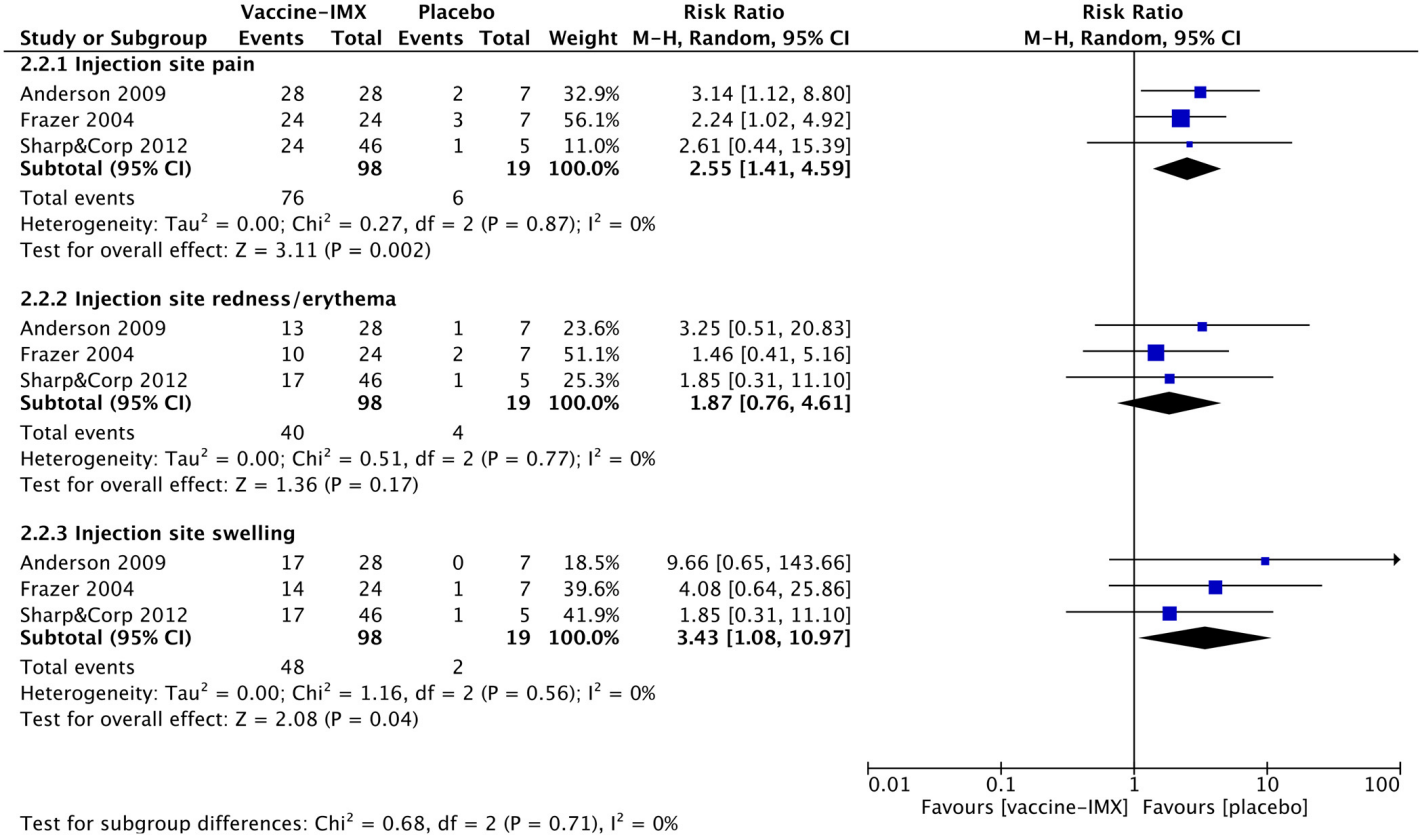
Meta-analysis of the reported local adverse events observed in non-healthy subjects receiving ISCOMATRIX-adjuvanted vaccines or placebo.

The reported local AEs were usually associated with injection site pain, redness/erythema and swelling. Individual studies reported also other local AEs such as warmth, bruising, injection site hematoma and injection site pruritus. Trials of Anderson *et al*. [47] and Frazer *et al*. [48] specified that the proportion of subjects experiencing an injection site reaction (including pain, swelling, warmth, redness and bruising) or injection site pain was greater in the groups receiving ISCOMATRIX-adjuvanted vaccines than in the group of subjects receiving placebo. In general, the observed local AEs were of mild to moderate intensity. The meta-analysis showed that there was no association between the exposure to the ISCOMATRIX-adjuvanted vaccines and the incidence of local redness/erythema (pooled RR 1.87, 95% CI 0.76–4.61). However, the ISCOMATRIX-adjuvanted vaccines significantly increased the likelihood of experiencing the injection site pain (pooled RR 2.55, 95% CI 1.41–4.59, p = 0.002) and swelling (pooled RR 3.43, 95% CI 1.08–10.97, p = 0.04) than placebo (Fig 5). Of note, the risk for injection site pain is approximately 1,6 times less for subjects receiving ISCOMATRIX-adjuvanted vaccines compared to subjects receiving QS-21-adjuvanted vaccines (pooled RR 2.55 vs. pooled RR 4.11). In contrast, the risk for injection site swelling increases two-fold in subjects receiving ISCOMATRIX-adjuvanted vaccines (pooled RR 3.43 vs. pooled RR 1.75).

#### Discontinuations due to AEs

Anderson *et al*. [47] reported that no participants withdrew due to the AEs. Frazer *et al*. [48] reported that three women who received ISCOMATRIX-adjuvanted vaccine did not complete the treatment because of AEs (12.5%, 95% CI 4.3–31%). The AEs that caused discontinuation were not specified. Besides, no discontinuation in the placebo group (0%, 95% CI 0–35%) was reported from the same study. Sharp&Corp [49] reported that 15.2% (95% CI 7.6–28.2%) subjects who received vaccine adjuvanted with ISCOMATRIX, 0% (95% CI 0–22.8%) subjects who received 16 μg ISCOMATRIX alone and 0% (95% CI 0–43.4%) subjects who received placebo discontinued the study due to the AEs (AEs not specified). Similar to what we observed from the QS-21-adjuvanted vaccine trials, more individuals in the ISCOMATRIX-adjuvanted vaccine group discontinued the treatment compared to the placebo group.

### Saponin-adjuvanted vaccine versus placebo

In order to study the general effect of saponin adjuvantation on the safety and tolerability of tested vaccines, we performed meta-analysis on the reported AEs from all nine eligible studies.

**Fig 6:**
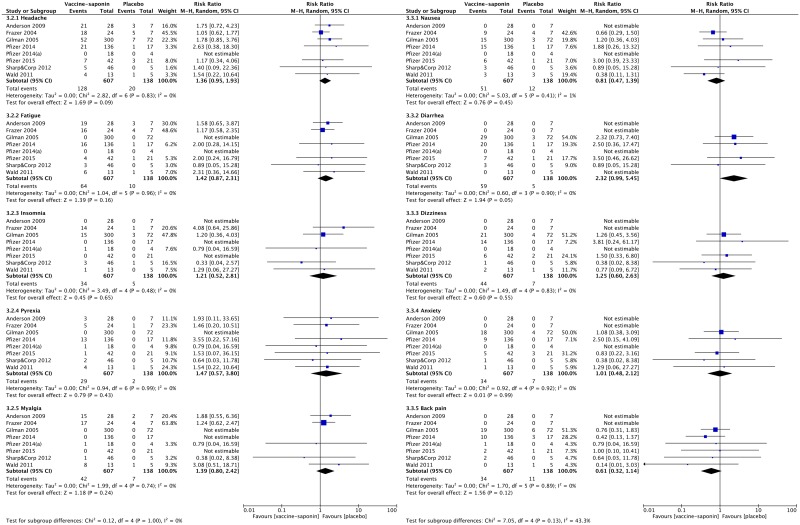
Meta-analysis of the reported systemic adverse events observed in non-health subjects receiving saponin-adjuvanted vaccines or placebo.

In the case of frequently reported systemic AEs, we were able to combine the dichotomous data on the number of non-healthy subjects experiencing headache, fatigue, insomnia, pyrexia, myalgia, nausea, diarrhea, dizziness, anxiety and back pain after immunization with QS-21- or ISCOMATRIX-adjuvanted vaccines (Fig 6). The meta-analysis showed that there was a trend towards a higher risk of systemic AEs in patients receiving saponin-adjuvanted vaccine compared to those receiving placebo, although the difference was not statistically significant (e.g. headache: pooled RR 1.36, 95% CI 0.95–1.93, p = 0.09; diarrhea: pooled RR 2.32, 95% CI 0.99–5.45, p = 0.05).

**Fig 7:**
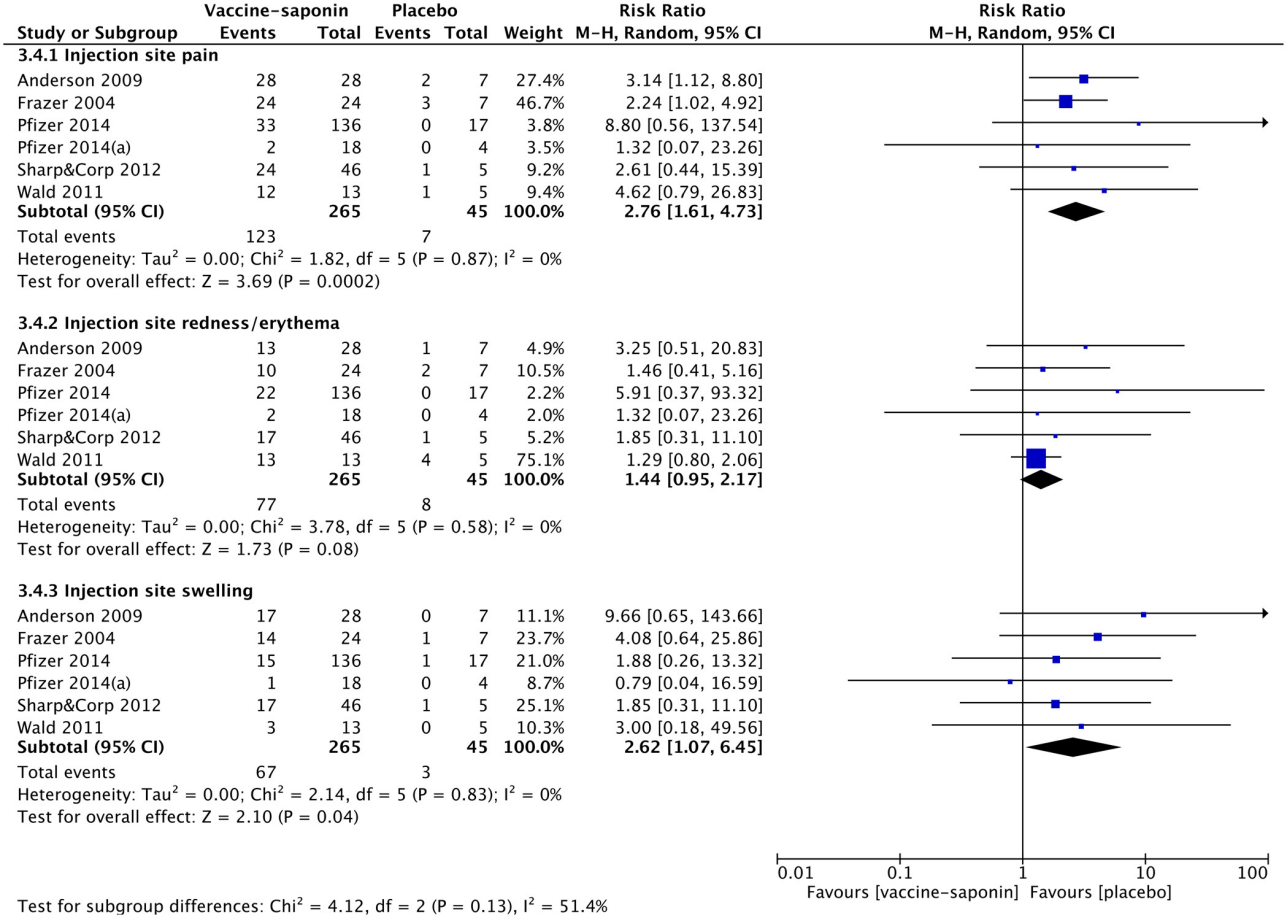
Meta-analysis of the local adverse events observed in non-healthy subjects receiving saponin-adjuvanted vaccines or placebo.

When combining the data on the frequently reported local AEs from all selected trials, the performed meta-analysis confirmed that immunization of non-healthy subjects with saponin-adjuvanted vaccines increased the risk for injection site pain (pooled RR 2.76, 95% CI 1.61–4.73, p = 0.0002) and injection site swelling (pooled RR 2.62, 95% CI 1.07–6.45, p = 0.04), when compared to placebo (Fig 7). Furthermore, the results showed a trend towards an increased risk for injection site redness/erythema in patients receiving saponin-adjuvanted vaccine compared to those receiving placebo, although it was not statistically significant (pooled RR 1.44, 95% CI 0.95–2.17, p = 0.08).

## Discussion

The objective of this meta-analysis was to assess the safety and tolerability of vaccines containing QS-21 and new saponin adjuvant formulations such as ISCOM and ISCOMATRIX. References were included if they reported on a RCT of vaccines with saponin adjuvants (QS-21, ISCOM, ISCOMATRIX, or Matrix-M^™^) including a placebo control group and reporting information regarding safety and/or tolerability. The effect of saponin-adjuvantation was evaluated by the RR with 95% CI for systemic and local AEs. Reports on SAEs and the number of subjects who discontinued treatment due to the AEs were discussed descriptively. We identified three studies reported on ISCOMATRIX-adjuvanted vaccines and six studies on vaccines adjuvanted with QS-21. All nine studies included adult (≥ 18 years) non-healthy subjects. Overall, we included in the meta-analysis 98 subjects receiving ISCOMATRIX-adjuvanted vaccines, 510 subjects receiving QS-21-adjuvanted vaccines and 138 receiving placebo. Eight of the nine trials have a Jadad score of 3 or 4 indicating an adequate trial quality.

SAEs were reported by five studies [41,43–46] on QS-21-adjuvanted vaccines and one study on vaccines adjuvanted with ISCOMATRIX (Sharp&Corp [49]). However, none of the observed SAEs were considered to be related to the use of the saponin adjuvants. The majority of SAE cases described by Gilman *et al*. [41] were associated with encephalitis. The authors linked the addition of polysorbate-80 to the vaccine formulation with the occurrence of the SAEs. Polysorbate-80 is an emulsifier that helps to improve the product stability, and was previously shown to be involved in the development of the inflammatory reaction. According to Gilman *et al*. [41], the addition of polysorbate-80 may have caused a greater exposure of antigenic epitopes, which might lead to an inflammatory T-cell response. All reported cases of encephalitis occurred mainly in the antibody non-responders and are reviewed in detail by Orgogozo *et al* [53]. Of note, none of four Pfizer trials in subjects with Alzheimer’s disease observed any cases of encephalitis. The SAEs reported by Sharp&Corp [49] were unlikely associated with the use of ISCOMATRIX as an adjuvant due to the fact that SAEs were not more frequent in the recipients of ISCOMATRIX-adjuvanted vaccine compared to those received placebo or the antigen alone.

Based on the performed meta-analysis, none of the reported systemic AEs were significantly increased upon the administration of ISCOMATRIX-adjuvanted vaccines. In the case of QS-21-adjuvanted vaccines, patients experienced significantly more cases of diarrhea compared to placebo. Most of the systemic AEs observed across the included studies were of mild to moderate intensity and of short duration. When we combined the systemic AEs from all the selected studies to evaluate the general effect of saponin adjuvantation on the safety and tolerability of the tested vaccines, we observed that none of the frequently reported systemic AEs were significantly increased upon the use of saponin-adjuvanted vaccines. In general, the relative risks of the reported systemic AEs from the pooled saponin studies are higher than those observed from the ISCOMATRIX-specific studies, but lower than those observed from the QS-21-specific studies.

The performed meta-analysis further showed that both QS-21- and ISCOMATRIX-adjuvanted vaccines are associated with a higher risk for injection site pain, although the estimated risk is twice lower for ISCOMATRIX-adjuvanted vaccines. On the other hand, the risk for injection site swelling is only increased upon the use of the vaccines containing ISCOMATRIX-adjuvanted vaccines. Although the use of QS-21-adjuvanted vaccines also resulted in a trend towards a higher risk for injection site swelling, the risk was not statistically significant and was 1.6 times less when compared to ISCOMATRIX-adjuvanted vaccines. When we pooled the data on reported local AEs from all selected trials, the meta-analysis confirmed that the use of saponin-adjuvanted vaccines significantly increased the likelihood of experiencing both injection site pain and swelling. This might provide a possible reason for the observation that generally more treatment discontinuations due to the AEs were reported from the saponin-adjuvanted vaccine recipients than control group.

The safety and tolerability profile of QS-21- and ISCOMATRIX-adjuvanted vaccines revealed by the performed meta-analysis in non-healthy subjects is similar to that observed in healthy volunteers. In healthy subjects, no vaccine related SAEs were observed receiving QS-21- or ISCOMATRIX-adjuvanted vaccines [26,39,54,55]. However, these trials conducted in healthy subjects often reported higher incidence of systemic AEs in saponin-adjuvanted vaccine study group than in placebo or an active control group. The systemic AEs reported in healthy subjects were mild to moderate in intensity, self-limiting and of short duration. Local AEs observed in healthy volunteers included local pain, redness and induration. The injection site pain was often moderate to severe and was more frequently reported by QS-21-adjuvanted vaccine recipients than those receiving placebo [54,55]. The study of Waite *et al*. [40] further confirmed that the presence of QS-21 in the injected formulation is associated with the injection site pain. Interestingly, McKenzie *et al*. [39] stated that the incidence of local and systemic AEs using ISCOMATRIX vaccines is similar to that published for other adjuvanted vaccines (e.g., AS04, aluminum containing adjuvant).

The selected RCTs aimed to assess not only the safety and tolerability of the study vaccines, but also the immunogenicity. To be able to determine the added immunogenicity value of an adjuvant, we need to compare the immune responses elicited by the adjuvanted study vaccines with non-adjuvanted vaccines (i.e. antigen-adjuvant vs. antigen alone). Only one out of three trials on ISCOMATRIX-adjuvanted vaccines [49] and four out of six trials on QS-21-adjuvanted vaccines [42–45] included appropriate study groups. Moreover, the high heterogeneity in immune parameters reported by different trials, such as the immune factors analyzed (i.e. antibody responses or cellular immune responses), the assays performed (i.e. enzyme-linked immunosorbent assay (ELISA) or enzyme-linked immunospot (ELISPOT)), the units used (i.e. geometric mean titer or mean fold-increase of a specific antibody response) and the time-frame of the analysis restrains us from performing meta-analysis on the immunological benefit of saponin adjuvants. However, the immune boosting effect of saponin adjuvants can be confirmed by the data reported from five of the selected trials [42–45,49]. One should consider that the immunological benefit of the saponin adjuvants should be weighed against the potential risk of adverse events. The significant increase in the incidence of injection site pain and swelling upon the immunization with saponin-adjuvanted vaccines might prevent the use of such adjuvants in routine immunizations, especially in the case of prophylactic vaccines.

The results of the meta-analysis should be interpreted with caution due to the several limitations. To have direct information about the safety and tolerability of adjuvants, the adjuvanted test vaccines should be compared to an active control group (immunization with the antigen alone or antigen with licensed adjuvants). Although there were five eligible RCTs [42–45,56,57] on QS-21-adjuvanted vaccines with an active control group (antigen alone), we identified only one eligible RCT on ISCOMATRIX-adjuvanted vaccine [49]. Due to the limitation, AEs reported from the saponin-adjuvant vaccine group were compared with those from the placebo group in our meta-analysis, which made the actual causes of AEs associated with the use of saponin-adjuvanted vaccines unidentifiable. In addition, the number of the included clinical trials meeting the inclusion criteria was limited. Furthermore, the number of subjects recruited to each of these trials was generally small, especially for the control groups. These contribute to the wide confidence intervals, and decrease the statistical power to detect statistically significant differences between the treatment groups. Due to the different settings of the trials included in the meta-analysis, we chose random-effects model for the meta-analysis, which further widens the confidence intervals. Last but not least, differences in the reporting method of observed AEs and the classification of AEs limit the possibility for including respectively more trials for the meta-analysis or perform meta-analysis on other reported AEs among the included studies.

## Conclusions

No major safety concern was identified for both ISCOMATRIX-adjuvanted vaccines and vaccines containing QS-21 based on the reported SAEs. Most AEs reported by non-healthy subjects in the nine selected trials were generally mild to moderate, self-limiting and of short duration. The performed meta-analysis showed that the use of QS-21-adjuvanted vaccines resulted in a statistically significant increase in the incidence of diarrhea when compared to placebo, while no systemic AEs were found to be associated with the use of ISCOMATRIX-adjuvanted vaccines. Both QS-21- and ISCOMATRIX-adjuvanted vaccines were associated with a higher incidence of injection site pain. The observed elevated risk for local pain was lower for the vaccines containing ISCOMATRIX. On the other hand, an increased incidence of injection site swelling was only observed from the use of ISCOMATRIX-adjuvanted vaccines. Furthermore, the pooled analysis on ISCOMATRIX- and QS-21-adjuvanted vaccines further confirmed that subjects receiving a saponin-adjuvanted vaccine experienced significantly more injection site pain and swelling when compared to placebo. In addition, for both adjuvants the number of subjects who discontinued treatment was higher in the group of subjects receiving the adjuvanted vaccine than in the placebo group. Our results indicate that the use of ISCOMATRIX results in a better systemic tolerability profile when compared to the use of QS-21. However, no better local tolerance was observed for ISCOMATRIX-adjuvanted vaccines in immunized non-healthy subjects. The relatively small number of published studies, however, limited our ability to calculate robust estimates for other AEs and to draw strong conclusions on the effects of QS-21- and ISCOMATRIX-adjuvanted vaccines. Therefore, further studies are needed, particularly with properly defined and reported safety outcomes and including an active control group, to better evaluate the risks of saponin adjuvanted vaccines.

## Supporting Information

S1 Appendix Search strategy. (PDF)

S2 Appendix PRISMA Checklist. (PDF)
