# Supplementary material for: Meta-Analysis on Randomized Controlled Trials of Vaccines with QS-21 or ISCOMATRIX Adjuvant: Safety and Tolerability
Source: PLoS One. 2016 May 5;11(5):e0154757. doi: 10.1371/journal.pone.0154757 (PMC4858302; doi:10.1371/journal.pone.0154757)
Supplement: S1 Appendix — (PDF) [file pone.0154757.s001.pdf]

S1 Table. Search strategy

Search strategy Medline (Ovid) 04.03.2016

Set	*Search term*	*Items found*
**Concept #1**		
1	qs-21.af.	243
2	qs21.af.	78
3	iscoms/	328
4	iscomatrix.af.	80
5	iscom-matrix.af.	56
6	matrix-m.af.	3652
7	adjuvant.af.	121497
8	6 and 7	30
9	1 or 2 or 3 or 4 or 5 or 8	741
**Concept #2**		
10	clinical trial.pt.	496894
11	exp clinical trial/	724380
12	(clinic$ adj25 trial$).tw.	275052
13	((singl$ or doubl$ or trebl$ or tripl$) adj (mask$ or blind$)).tw.	130356
14	random$.tw	717353
15	random allocation/	85593
16	placebos/	33035
17	placebo$.tw.	160396
18	research design/	87088
19	or/10-18	1408904
20	animal/ not human/	4159388
21	19 not 20	1462184
22	9 and 21	159

Search strategy Embase 04.03.2016

Set	*Term*	*Items found*
**Concept #1**		
1	‘qs21’	103
2	‘qs 21’	782
3	‘iscoms’	342
4	‘iscoms’/exp	608
5	‘iscomatrix’	160
6	‘iscom-matrix’	60
7	‘matrix-m’	683
8	OR/1-7	2291
**Concept #2**		
9	‘clinical trial’:ti	35909
10	‘clinical trial’/exp	1061457
11	‘clinical trial’	1177385
12	(singl* OR doubl* OR trebl* OR tripl*) AND (mask* OR blind*)	257466
13	random*	1204262
14	‘randomization’/exp	68683
15	‘Placebo’/exp	282850
16	Placebo*	365946
17	‘experimental design’/exp	12258
18	‘control group’/exp	90435
19	OR/9-18	2146461
20	19 AND [humans]/lim	1799726
21	8 AND 20	578

Search Cochrane Library 04.03.2016

Search term	***Items found***
qs21 or qs-21 or iscoms or iscom or iscomatrix or iscom-matrix or matrix-m :ti,ab,kw<br>(Word variations have been searched)	65

Search Clinicaltrials.gov 04.03.2016

Search term	***Items found***
qs21 OR qs-21 OR iscoms OR iscom OR iscomatrix OR (matrix-m AND adjuvant)	11

Applied filters: recruitment – closed studies; exclude studies with unknown status;
study results – studies with results
